# Identification of *COL4A4* variants in Chinese patients with familial hematuria

**DOI:** 10.3389/fgene.2022.1064491

**Published:** 2023-01-09

**Authors:** Yanan Gao, Lamei Yuan, Jinzhong Yuan, Yan Yang, Jiangang Wang, Yong Chen, Hao Zhang, Yinze Ai, Hao Deng

**Affiliations:** ^1^ Health Management Center, The Third Xiangya Hospital, Central South University, Changsha, China; ^2^ Center for Experimental Medicine, The Third Xiangya Hospital, Central South University, Changsha, China; ^3^ Disease Genome Research Center, Central South University, Changsha, China; ^4^ Department of Nephrology, The Third Xiangya Hospital, Central South University, Changsha, China; ^5^ Department of Neurology, The Third Xiangya Hospital, Central South University, Changsha, China; ^6^ National Health Committee Key Laboratory of Birth Defects for Research and Prevention, Hunan Provincial Maternal and Child Health Care Hospital, Changsha, China

**Keywords:** benign familial hematuria, Alport syndrome, collagen type IV alpha 4 chain gene, whole exome sequencing, splicing variant

## Abstract

**Background:** Benign familial hematuria and Alport syndrome are common causes of familial hematuria among children and young adults, which are attributable to variants in the collagen type IV alpha chain genes, *COL4A3*, *COL4A4*, or *COL4A5*. The study was conducted to identify the underlying genetic causes in patients with familial hematuria.

**Methods:** Two unrelated Han-Chinese pedigrees with familial hematuria were recruited for this study. Whole exome sequencing was combined with *in silico* analysis to identify potential genetic variants, followed by variant confirmation by Sanger sequencing. Reverse transcription, PCR, and Sanger sequencing were performed to evaluate the effect of the detected splicing variant on mRNA splicing.

**Results:** A novel heterozygous splicing c.595-1G>A variant and a known heterozygous c.1715G>C variant in the collagen type IV alpha 4 chain gene (*COL4A4*) were identified and confirmed in patients of pedigree 1 and pedigree 2, respectively. Complementary DNA analysis indicated this splicing variant could abolish the canonical splice acceptor site and cause a single nucleotide deletion of exon 10, which was predicted to produce a truncated protein.

**Conclusions:** The two *COL4A4* variants, c.595-1G>A variant and c.1715G>C (p.Gly572Ala) variant, were identified as the genetic etiologies of two families with familial hematuria, respectively. Our study broadened the variant spectrum of the *COL4A4* gene and explained the possible pathogenesis, which will benefit clinical management and genetic counseling.

## Introduction

The familial hematuria of glomerular origin is usually due to a group of genetically and phenotypically heterogeneous disorders, such as benign familial hematuria (BFH), Alport syndrome (AS), glomerulopathy with fibronectin deposits (GFND), and C3 glomerulonephritis (C3GN) (Deltas et al., 2013). The common causes of familial hematuria among children and young adults are BFH and AS (Kashtan, 2009; Gale, 2013). BFH (OMIM 141200) is usually a non-progressive autosomal dominant renal disorder with a penetrance of 70% (Blumenthal et al., 1988; Lemmink et al., 1996; Savige et al., 2013), which is mainly characterized by persistent or recurrent glomerular hematuria, diffuse thinning of the glomerular basement membrane (GBM), and normal renal function (Slajpah et al., 2007). BFH is usually referred to as thin basement membrane nephropathy (TBMN) (Marcocci et al., 2009; Deltas et al., 2013; Gale, 2013), but TBMN is no longer considered benign as some can develop into end-stage renal disease (ESRD) in adults (Dische et al., 1985; Naylor et al., 2021). The frequency of this condition is estimated to be at least 1.0% in the worldwide population (Buzza et al., 2001; Chan and Gale, 2015), 5.2% in a population with microscopical examination and morphometry of GBM (Dische et al., 1990), and 15.5%–26% in children with hematuria and renal biopsy (Piqueras et al., 1998; Roth et al., 2001). It seems to be more prevalent in females than in males (Buzza et al., 2001; Savige et al., 2003). BFH is often attributable to heterozygous variants in the collagen type IV alpha 3 chain gene (*COL4A3*) or the collagen type IV alpha 4 chain gene (*COL4A4*), arranging head to head on chromosome 2 (Boye et al., 1998; Tazón Vega et al., 2003). AS most often manifests hematuria, proteinuria, and progressive renal failure, sometimes associated with sensorineural hearing loss and ocular abnormalities (Hudson et al., 2003), and the renal pathology is characterized by irregular thickening, thinning, and lamellation of GBM (Savige et al., 2013; Kamiyoshi et al., 2016). The *COL4A3*/*COL4A4* heterozygous variants were also reported to cause autosomal dominant Alport syndrome (ADAS, OMIM 104200) (Boye et al., 1998), while homozygous or compound heterozygous variants were responsible for the autosomal recessive Alport syndrome (ARAS) (Longo et al., 2002; Voskarides et al., 2007). The heterozygous carriers of the ARAS family can present manifestations like BFH (Heidet et al., 2001; Savige et al., 2003). X-linked AS (XLAS), the most common form, accounts for about 85% of AS patients, which is ascribed to variants in the collagen type IV alpha 5 chain gene (*COL4A5*) (Slajpah et al., 2007; Nabais Sá et al., 2015). Compared with ARAS and XLAS, ADAS is relatively milder and slowly progressive with rare extrarenal manifestations (Kamiyoshi et al., 2016). Given the heterotrimeric association of type IV collagen α3/α4/α5 chain in the GBM and the overlapping clinical symptoms, a spectrum ranging from totally asymptomatic or isolated microscopic hematuria to proteinuria, up to chronic renal failure (CRF) and ESRD (Nabais Sá et al., 2015; Furlano et al., 2021; Kashtan, 2021), it is rational to consider BFH and AS as an entity and to term them collectively as collagen IV-related nephropathies (Tazón Vega et al., 2003; Pieri et al., 2014). BFH is considered to be the mildest end in the continuous spectrum, and the most severe end is represented by ARAS (Lemmink et al., 1996; Nieuwhof et al., 1997; Xu et al., 2016). However, BFH can resemble early AS, especially ADAS, in initial clinical presentation and the electron-microscopic features of the GBM, rendering the exact diagnosis notoriously difficult (Badenas et al., 2002; Gross et al., 2003; Voskarides et al., 2007). A complete investigation of family history, careful clinical evaluation, long-term specialist follow-up, and available renal biopsy analysis are vital in avoiding misdiagnosis or missed diagnosis (Kashtan, 2021). This study aimed to identify the genetic etiologies of familial hematuria in two unrelated Han-Chinese families and to elucidate possible morbigenous mechanisms.

## Materials and methods

### Subjects and clinical evaluations

**FIGURE 1:**
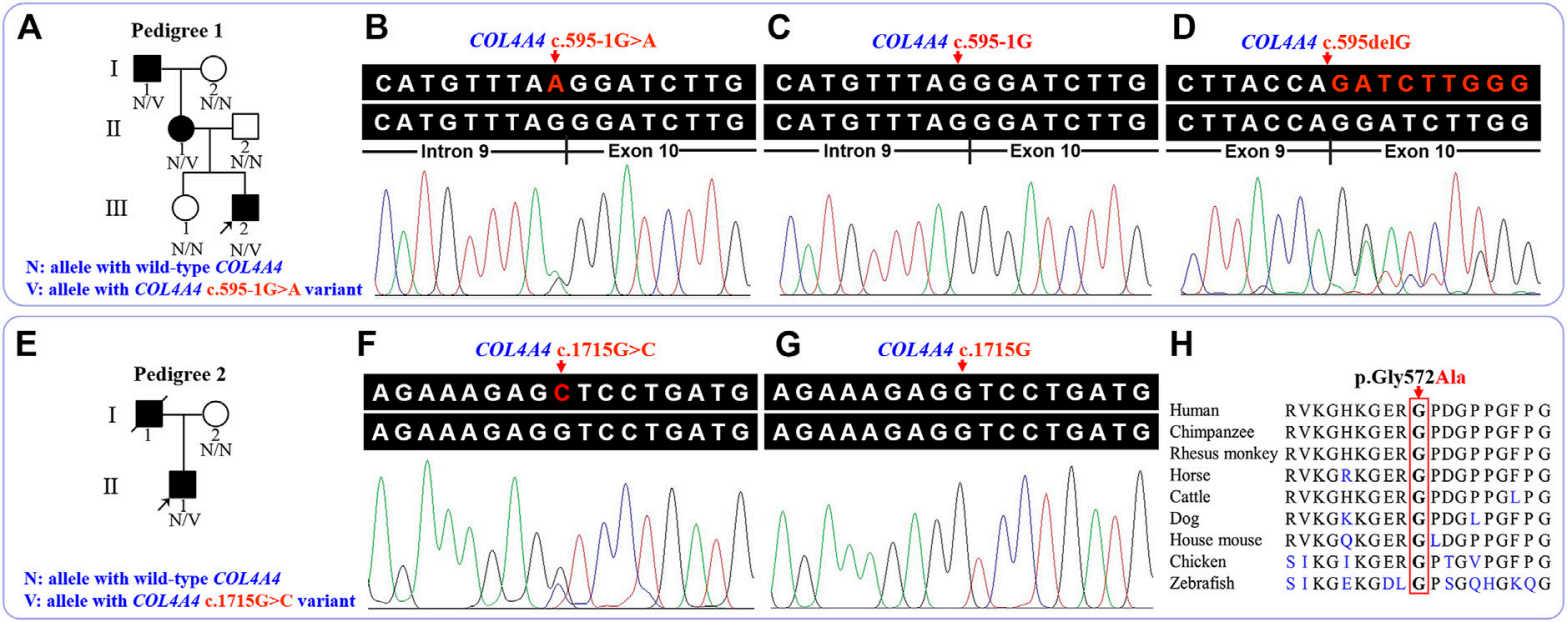
Pedigree and sequence analysis of the two unrelated Han-Chinese families with familial hematuria. **(A)** Pedigree 1. Squares and circles represent males and females, respectively. The fully shaded symbol indicates the affected individual. Arrow indicates the proband. **(B)** The genomic sequence with heterozygous *COL4A4* c.595-1G>A variant (III:2 in pedigree 1). **(C)** The wild-type *COL4A4* genomic sequence (II:2 in pedigree 1). **(D)** Analysis of complementary DNA revealed a deletion of the first base of exon 10 (III:2 in pedigree 1). **(E)** Pedigree 2. Arrow indicates the proband. The slash indicates deceased individual. **(F)** The genomic sequence with heterozygous *COL4A4* c.1715G>C variant (II:1 in pedigree 2). **(G)** The wild-type *COL4A4* genomic sequence (I:2 in pedigree 2). **(H)** Conservation analysis of the COL4A4 p.Gly572 residue. *COL4A4*, the collagen type IV alpha 4 chain gene.

Members of two Han-Chinese pedigrees were recruited from the Third Xiangya Hospital of Central South University, China (Figure 1). Detailed audiological examinations and ophthalmologic assessments were performed, including pure-tone audiometry, acoustic immittance, otoacoustic emissions, auditory brainstem response, slit-lamp evaluation, fundus examination, and optical coherence tomography. Peripheral blood samples were collected from four patients and four asymptomatic family members of two pedigrees, together with available clinical information. Written informed consents were obtained from all participating individuals. The entire study complied with the Declaration of Helsinki guidelines and was approved by the Institutional Review Board of the Third Xiangya Hospital of Central South University, China.

### DNA extraction, exome capture, and sequencing

Whole exome sequencing (WES) was conducted on the proband and their parents of pedigree 1, and the proband of pedigree 2. Genomic DNA (gDNA) of participating individuals was extracted from peripheral venous blood lymphocytes following the manufacturer’s protocol (Huang et al., 2021; Xiong et al., 2021). The qualified gDNA was fragmented randomly and then processed for end-repairing, A-tailing, and adaptor ligation. These adapter-ligated fragments were amplified by polymerase chain reactions (PCR) followed by purification and exome array hybridization. After further enrichment and purification, the captured libraries were sequenced on the high-throughput sequencing platforms: Illumina NextSeq500 (pedigree 1) and DNBSEQ (pedigree 2).

### Variant analysis and Sanger sequencing

After filtering the raw data from the sequencing platform, data processing including sequence alignment, variant calling, annotation, and analysis was performed according to previously described criteria (Wu et al., 2016; Wu et al., 2019), as detailed in the Supplementary Methods.

The potential pathogenic variants identified in patients were further tested in participating individuals *via* Sanger sequencing. The following are the sequences of primers: 5′-GCT​GGT​GGC​TGT​GAT​TTC​TT-3′ and 5′-CAC​CTG​TGT​CTG​ACC​CAA​AA-3′ for detecting the potential variant in the pedigree 1, 5′-CCA​ACC​CAG​AAT​CAA​GGT​CA-3′ and 5′-TCC​TGG​ATC​CCC​TTT​TTC​TC-3′ for detecting potential variant in pedigree 2.

### RNA isolation, complementary DNA synthesis, and PCR analysis

To analyze the effect of identified splicing variant, total RNA was extracted from the peripheral blood lymphocytes of the patients (I:1, II:1, and III:2) in pedigree 1 using the TRIzol reagent (Invitrogen, Carlsbad, CA, United States). The complementary DNA (cDNA) was synthesized by reverse transcription using the First Strand cDNA Synthesis Kit (Toyobo, Japan), and further amplified by PCR using the specifically designed primers: 5′-TGG​GGA​AAA​GGG​AGA​AAA​AG-3′ and 5′-ACC​TTG​CTG​ACC​AAC​CTC​AC-3′. PCR products were checked by agarose gel electrophoresis and then analyzed on the ABI 3730XL DNA sequencer (ABI, Carlsbad, CA, United States).

### Conservative analysis and variant evaluation

The protein sequence alignment among nine species was completed by the NCBI Basic Local Alignment Search Tool (BLAST, https://blast.ncbi.nlm.nih.gov/BlastAlign.cgi). The American College of Medical Genetics and Genomics (ACMG) guidelines for interpreting sequence variants were utilized to classify the identified variants (Richards et al., 2015; Savige et al., 2021).

### Quantification of nonsense-mediated decay by Sanger sequencing

Nonsense-mediated decay (NMD) was determined by sequencing analysis of *COL4A4* cDNA from patients I:1, II:1, and III:2 of pedigree 1, and by comparing the areas under the peaks (AUP) of wild-type (WT) and mutant (MT) alleles. The AUP of the WT and MT specific-alleles were quantified by the ImageJ software v1.53a (NIH, United States), and the ratio of AUP of MT over corresponding WT was then calculated. Statistical analysis was performed using the Microsoft Excel 2016 software (Microsoft, Inc.) and GraphPad Prism v9.4.0 (GraphPad Software, Inc.) using Student’s *t* test. *p*<.05 was considered significant.

## Results

### Clinical data

The proband (III:2) of pedigree 1, a 15-year-old boy, was accidentally found to have microscopic hematuria during the physical examination for enrollment 3 years ago. The dysmorphic erythrocytes in the urinary sediment observed by phase-contrast microscopy supported the diagnosis of glomerular hematuria. Further investigation found that his mother (II:1) also had long-term microscopic hematuria, occasionally accompanied by trace proteinuria by routine tests. His 71-year-old grandfather (I:1) had microscopic hematuria persisting for an uncertain time. All three patients exhibit no signs of impairment in blood pressure or renal function. Audiological and ophthalmologic assessments of the proband and his mother demonstrated no signs of sensorineural hearing loss or ocular lesions.

**FIGURE 2:**
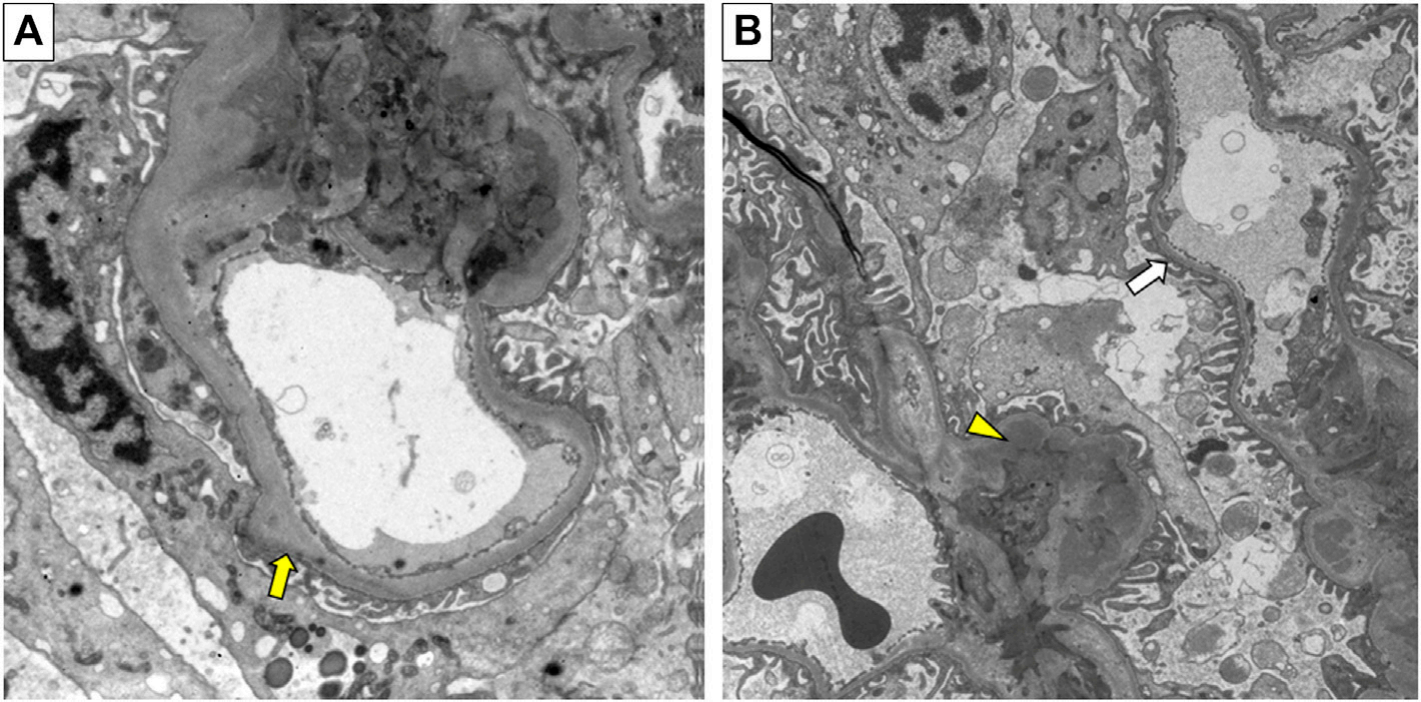
Electron micrograph of renal biopsy specimen from the proband (II:1) in pedigree 2. **(A)** Irregular thickening of the glomerular basement membrane (GBM) (yellow arrow). **(B)** Irregular thinning of the GBM (white arrow), and electron-dense deposits (yellow triangle). Original magnification 6500×.

The proband (II:1) of pedigree 2, a 30-year-old male, displayed microscopic hematuria and a mildly elevated serum creatinine level on routine physical examination. His serum creatinine fluctuated between 110 and 123 μmol/L (reference interval: 59–104 μmol/L) during 3 years of follow-up. Renal biopsy was performed in the Sichuan Provincial People’s Hospital half a year ago. Light microscopy showed segmental mild hyperplasia of mesangial cells and matrix. Immunofluorescence staining of IgM, IgA, C3, κ, and λ was positive, showing a massive pattern of deposition within the mesangium. On electron microscopy, there is segmental mild mesangial expansion with massive electron-dense deposits and segmental irregular thickening and thinning of the GBM **(** Figure 2 **)**. The audiological and ophthalmologic evaluations revealed no abnormalities. Clinical data of these patients were summarized in Table 1.

**TABLE 1:** Clinical data of patients with the *COL4A4* gene variant.

Items	Pedigree 1			Pedigree 2
	I:1	II:1	III:2	II:1
Sex	Male	Female	Male	Male
Age (years)	71	41	15	30
BMI^a^ (kg/m^2^)	27.7	20.6	22.4	21.5
Serum creatinine^b^ (µmol/L)	96	61	76	123
eGFR^c^ (ml/min/1.73 m^2^)	68	108	129	67
Microscopic hematuria	Trace	2+	1+	2+
Gross hematuria	No	No	No	No
Proteinuria	No	Trace	No	No
Hypertension	No	No	No	No
GBM changes by EM	NA	NA	NA	Irregular thickening and thinning
Sensorineural hearing loss	No	No	No	No
Ocular abnormalities	No	No	No	No

BMI, body mass index; eGFR, estimated glomerular filtration rate; GBM, glomerular basement membrane; EM, electron microscopy; NA, not available.

^a^ Normal weight, 18.5≤BMI<23; overweight, 23≤BMI<25; obesity, BMI≥25.

^b^ Reference interval for the test of serum creatinine is 57–111 μmol/L for males and 41–81 μmol/L for females in pedigree 1, and 59–104 μmol/L for males in pedigree 2, using distinct methods.

^c^ eGFR was calculated by the chronic kidney disease epidemiology collaboration (CKD-EPI) equation, and the reference interval is 56–122 ml/min/1.73 m^2^.

### WES and identification of pathogenic variants

The detailed WES data of four individuals are available in Supplementary Table 1. After database and *in silico* analysis screening, a novel heterozygous c.595-1G>A variant in the intron 9 of the *COL4A4* gene (NG_011592.1, NM_000092.5) was suggested as the potential pathogenic factor of patients in pedigree 1, and a known heterozygous c.1715G>C (p.Gly572Ala) variant in the exon 24 of the *COL4A4* gene was considered to be the potential disease-related variant of the proband in pedigree 2. The results of *in silico* analysis are presented in Table 2. After Sanger sequencing, the heterozygous splicing variant, c.595-1G>A, was identified in the patients I:1, II:1, and III:2 of pedigree 1, and the heterozygous missense variant c.1715G>C was confirmed in the proband II:1 of pedigree 2. These two variants were absent in the enrolled asymptomatic family members. For the reverse transcription PCR products, agarose gel electrophoresis and Sanger sequencing revealed no exon skipping or intron retention. Sanger sequencing indicated that the *COL4A4* c.599-1G>A substitution can abolish the intron 9 canonical splice acceptor site and introduce a new splice site, resulting in the loss of the first base of exon 10 during splicing, which was predicted to cause a frame shift and premature termination of translation (c.595delG, p.Gly199Aspfs*20). The COL4A4 glycine at position 572 (p.Gly572) is highly conserved across species (Figure 1). According to ACMG guidelines, the two variants were classified as “pathogenic”.

**TABLE 2:** Identification of the *COL4A4* gene variants in two pedigrees with familial hematuria.

Items		Variant 1	Variant 2
Exon		—	24
Intron		9	—
Nucleotide change		c.595-1G>A	c.1715G>C
Amino acid change		p.Gly199Aspfs*20	p.Gly572Ala
Genotype		Heterozygous	Heterozygous
Variant type		Splicing	Missense
dbSNP154 rs number		No	rs1446915781
Allele frequencies	1000G	No	No
	ESP6500	No	No
	gnomAD	No	4.01×10^−6^
	ChinaMAP	No	9.44×10^−5^
HGMD accession number		No	CM176102
ClinVar		No	Likely pathogenic
MutationTaster2021		Deleterious	Deleterious
SIFT		—	Damaging
PROVEAN		—	Deleterious
PolyPhen-2		—	Probably damaging
NetGene2 server		Destroy the acceptor site	—
BDGP NNSplice v0.9		Destroy the acceptor site	—
ACMG criteria applied		PVS1+PS3+PM2+PP1+PP3	PM1+PM2+PP2+PP3+PP5

dbSNP154, Single Nucleotide Polymorphism Database (version 154); rs, Reference SNP; 1000G, 1000 Genomes Project; ESP6500, Exome Sequencing Project 6500; gnomAD, Genome Aggregation Database; ChinaMAP, China Metabolic Analytics Project; HGMD, Human Gene Mutation Database; SIFT, Sorting Intolerant from Tolerant; PROVEAN, Protein Variation Effect Analyzer; PolyPhen-2, Polymorphism Phenotyping version 2; BDGP NNSplice, Berkeley Drosophila Genome Project Splice Site Prediction by Neural Network; ACMG, American College of Medical Genetics and Genomics; PVS, pathogenic very strong; PS, pathogenic strong; PM, pathogenic moderate; PP, pathogenic supporting.

### Quantification of NMD

**FIGURE 3:**
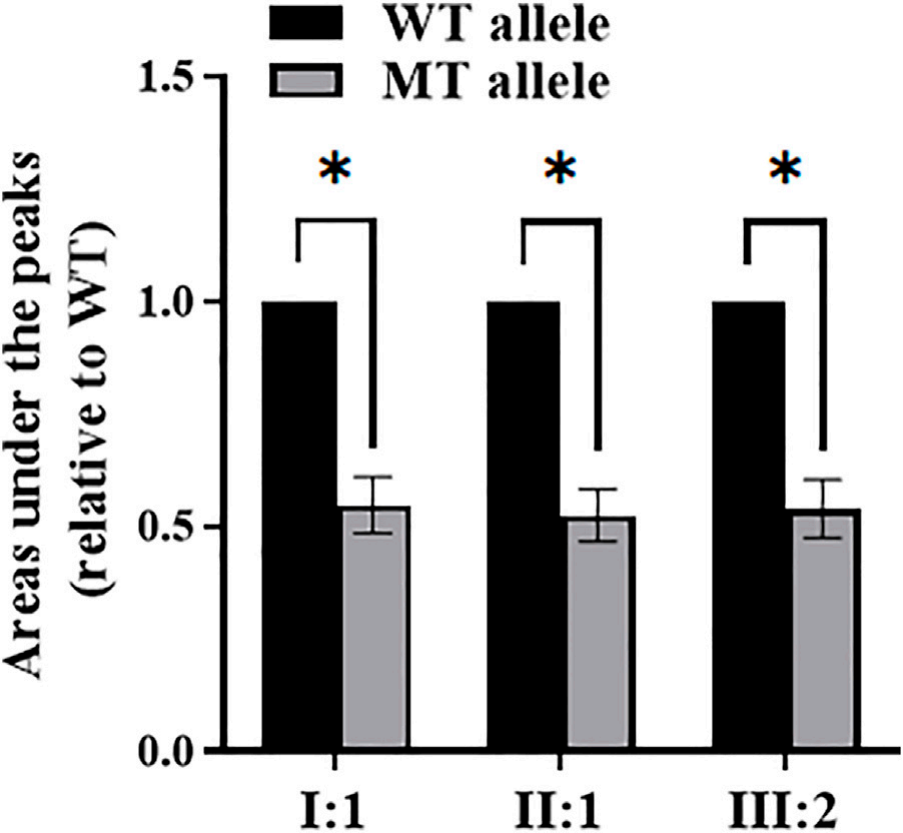
The areas under the peaks of wild-type (WT) and mutant (MT) alleles by sequencing analysis of *COL4A4* cDNA from patients I:1, II:1, and III:2 of pedigree 1. *Statistically significant differences (Student’s *t* test, *p*<.0001).

The differences between the AUP of the WT and MT alleles are statistically significant (*p*<.0001). The relative expression of the MT allele is approximately 50% lower than the WT allele by comparing the AUP of specific alleles in the cDNA sequencing chromatogram of these three patients (I:1, II:1, and III:2) in pedigree 1 (Figure 3).

## Discussion

**FIGURE 4:**
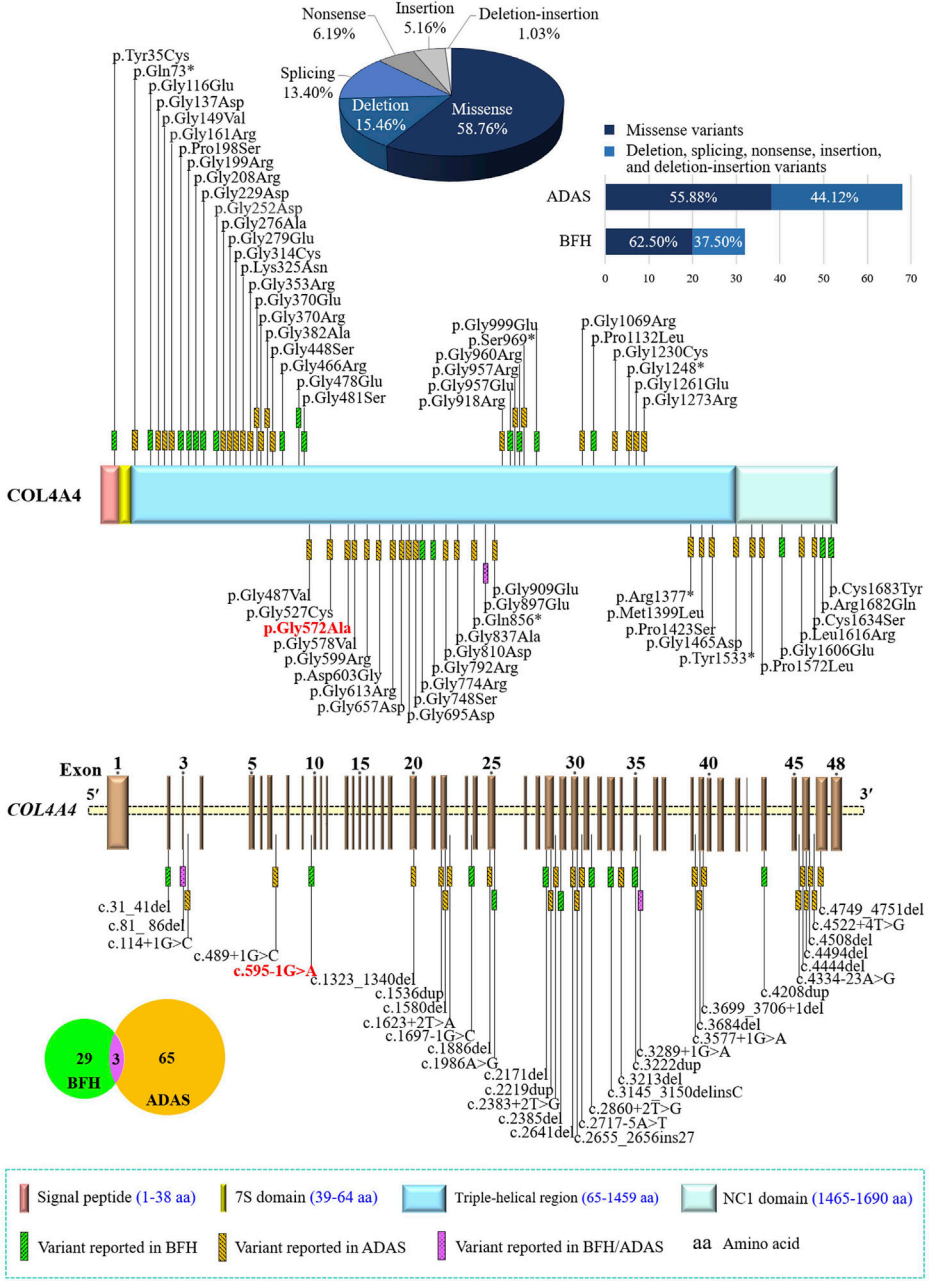
Statistics of the *COL4A4* gene variants related to benign familial hematuria (BFH) or autosomal dominant Alport syndrome (ADAS). Collagen type IV alpha 4 chain contains an N-terminal 7S domain, a long, central triple-helical region, and a C-terminal non-collagenous (NC1) domain. Variants identified in this study were indicated in bold and red. *COL4A4*, the collagen type IV alpha 4 chain gene.

The *COL4A4* gene (OMIM 120131), located at 2q36.3, contains 48 exons encoding the α4 chain of type IV collagen (Boye et al., 1998). Each chain contains an N-terminal 7S domain, a long, central, triple-helical collagenous domain of Gly-Xaa-Yaa repeats frequently interrupted by short non-collagenous regions, and a C-terminal non-collagenous (NC1) domain (Figure 4) (Boutaud et al., 2000; LeBleu et al., 2010). The six highly homologous chains of type IV collagen, namely α1(IV) to α6(IV), were encoded by six genes (*COL4A1* to *COL4A6*) (Hudson et al., 2003; Cicuéndez et al., 2021). In mammals, the existence of six distinct α(IV) chains could only assemble into three different trimers, namely α1α1α2(IV), α3α4α5(IV), and α5α5α6(IV) (LeBleu et al., 2010). During fetal development, collagen α1α1α2(IV) is ubiquitously distributed in all basement membranes (BMs) (Sand et al., 2013), while in the mature GBM and BMs of cochlea and eye, α3α4α5(IV) is the major component (Hudson et al., 2003; Suleiman et al., 2013; Savige et al., 2015). The pathogenic variants in any one of the three genes (*COL4A3*, *COL4A4*, and *COL4A5*) may interfere with the developmental switch, resulting in the persistent expression or compensatory increase of fewer cross-linked and more protease-susceptible α1α1α2(IV), which leads to BFH or AS (Suleiman et al., 2013; Clark et al., 2016; Xu et al., 2016). Currently, more than 400 *COL4A4* gene variants have been recorded in the Human Gene Mutation Database (HGMD), of which at least 29 and 65 variants are associated with BFH and ADAS, respectively, and three variants can cause both disorders. These different variants are widely distributed in the *COL4A4* gene without hot spots described, and the most frequent variants are glycine substitutions (46/97) (Figure 4). The frequency of nonsense, splicing, and frameshift variants in ADAS seems to be higher than that in BFH (44.12% vs. 37.50%), consistent with that these variants usually cause more severe phenotypes (Shang et al., 2019).

In this study, two variants in the *COL4A4* gene were identified in two unrelated Chinese pedigrees with familial hematuria. The heterozygous c.595-1G>A variant is completely co-segregated with the disorder phenotype within pedigree 1. The heterozygous c.1715G>C transversion detected in the proband of pedigree 2 has been previously reported in compound heterozygosity with variant c.-23T>G in a Chinese AS male patient, and both his sons with heterozygous c.-23T>G variant only presented isolated hematuria (Liu et al., 2017), supporting that the identified c.1715G>C variant may be pathogenic and seems to exert a pathogenic effect to result in a more severe renal phenotype. The absence of these two variants in the asymptomatic family members, the prediction of its deleterious effect by *in silico* programs, cDNA analysis, protein sequence alignment, and ACMG criteria evaluation suggested that they were likely disease-causing variants in these two pedigrees.

The splicing variant c.595-1G>A may induce NMD of the altered *COL4A4* mRNA or produce a truncated loss-of-function α4(IV) chain, consistent with similar studies (Schwarze et al., 2001; Davidson et al., 2007; Korstanje et al., 2014; Hashikami et al., 2018). The missense variant c.1715G>C (p.Gly572Ala) occurred in the first position of critical Gly-Xaa-Yaa repeats in the collagenous domain. The highly conserved glycine residue is the smallest amino acid that can fit tightly into the center of the triple helix structure of the α(IV) chain and is crucial to the helix formation (Deltas et al., 2013; Murray et al., 2014). Hence, both variants in the *COL4A4* gene may disrupt the normal synthesis of the α4(IV) chain and cause defective synthesis, assembly, deposition, or function of the α3α4α5(IV) network (Korstanje et al., 2014; Kashtan et al., 2018), interfering natural development process and leading to the abnormal GBMs (Clark et al., 2016; Naylor et al., 2021). Additionally, the poor association of abnormal α4(IV) with the α3(IV) and α5(IV) chain may leave more misfolded protein in the endoplasmic reticulum (ER), disrupt podocyte’s special secretory capacity, and lead to GBM defects (Pieri et al., 2014). These defective GBMs may partially allow the escape of erythrocytes into Bowman’s space, thereby triggering the onset of hematuria (Yoshikawa et al., 1984).

In pedigree 1, isolated microscopic hematuria, normal renal function, and absence of other signs related to AS in the proband, his mother, and grandfather, are more suggestive of a heterozygous *COL4A4-*related BFH than AS. In pedigree 2, given that the proband presented microscopic hematuria, mildly elevated serum creatinine level, and irregular thickening and thinning of the GBM, without signs of sensorineural hearing loss or ocular lesions, a diagnosis of early ADAS cannot be ruled out. The IgA deposits within the mesangium corresponding to IgA nephropathy could be due to the predisposition of mesangial IgA deposition in defective GBMs caused by *COL4A4* variant (Savige and Harraka, 2021). Usually, from clinical phenotype and renal biopsy, the coexistence of AS and IgA nephropathy is attributed to two independent causes. However, some variants in the *COL4A3*, *COL4A4*, or *COL4A5* gene seem to increase the susceptibility to IgA nephropathy, which is supported by IgA deposits occasionally observed in the AS patients and the *COL4A3*, *COL4A4*, or *COL4A5* gene variants reported in a minority of IgA nephropathy patients (Kamiyoshi et al., 2016; Li et al., 2020; Cambier et al., 2021). Based on the identified responsible variant, *COL4A4*-associated nephropathies, a sub-classification of the conditions in the two pedigrees, is strongly recommended. In this way, these similar or overlapping clinical manifestations could be attributed to a single disease spectrum rather than several distinct disease statuses. It would, therefore, be beneficial to risk assessment, disease management, and genetic counseling of diseased individuals.

Taken together, a novel c.595-1G>A variant and a known c.1715G>C (p.Gly572Ala) variant of the *COL4A4* gene were identified in two Chinese families with familial hematuria, respectively. WES has been proven to be a powerful tool for uncovering the genetic etiologies of heterogeneous disorders and identifying at-risk individuals, which will assist in early clinical diagnosis and accurate disease classification. Currently, there is a lack of evidence-based treatment for persistent microscopic hematuria (Xu et al., 2016), but it is of paramount importance to regularly monitor proteinuria development, renal function, and blood pressure every 1-2 years (Savige et al., 2003). Our findings broadened the variant spectrum of the *COL4A4* gene and may assist in reproductive risk counseling of these two families and improved medical care.

## Data Availability

The datasets presented in this article are not readily available because the data information is in a controlled state due to the national legislation, specifically the Ministry of Science and Technology of the People’s Republic of China. Data of this project can be accessed after an approval application by the China National GeneBank DataBase (CNGBdb). Please refer to CNGBdb: https://db.cngb.org/, or email: CNGBdb@cngb.org for detailed application guidance. The project accession code CNP0003636 should be included in the application.
