# Supplementary material for: Identification of COL4A4 variants in Chinese patients with familial hematuria
Source: Front Genet. 2023 Jan 9;13:1064491. doi: 10.3389/fgene.2022.1064491 (PMC9868811; doi:10.3389/fgene.2022.1064491)
Supplement: Supplementary file 1 [file DataSheet1.pdf]

Supplementary Material

1 Supplementary Methods

After filtering the raw data from the sequencing platforms, the acquired clean data were aligned to the human reference genome (GRCh37/hg19) via Burrows-Wheeler Aligner (BWA). Genome Analysis Toolkit (GATK) was utilized to guide the variant calling. The identified variants were analyzed using the ANNOVAR or SnpEff annotation tools. Databases like the Single Nucleotide Polymorphism database (dbSNP, version 154, http://www.ncbi.nlm.nih.gov/SNP), the 1000 Genomes Project (1000G, https://www.internationalgenome.org/), Exome Sequencing Project 6500 (ESP6500, https://evs.gs.washington.edu/EVS/), Genome Aggregation Database (gnomAD, http://gnomad-sg.org/), China Metabolic Analytics Project (ChinaMAP, http://www.mbiobank.com/), Human Gene Mutation Database (HGMD, https://www.hgmd.cf.ac.uk/ac/index.php), and the ClinVar (http://www.ncbi.nlm.nih.gov/clinvar) were used for screening potential pathogenic variants. Pathogenicity of the variants was further predicted by MutationTaster2021, Sorting Intolerant from Tolerant (SIFT), Protein Variation Effect Analyzer (PROVEAN), Polymorphism Phenotyping version 2 (PolyPhen-2), NetGene2 server, and Berkeley Drosophila Genome Project (BDGP) Splice Site Prediction by Neural Network (v0.9) (Hebsgaard et al., 1996; Reese et al., 1997; Adzhubei et al., 2013; Choi and Chan, 2015; Vaser et al., 2016; Steinhaus et al., 2021; Yuan et al., 2021; Yu et al., 2022).

2 Supplementary Tables

Supplementary Table 1 Summary of whole exome sequencing data

Samples	Pedigree 1			Pedigree 2
	II:1	II:2	III:2	II:1
Raw reads	102,953,438	109,214,820	101,659,130	13,145,619,300
Clean reads	102,908,734	109,163,758	101,610,848	86,289,328
Map ratio*	99.42%	99.27%	99.27%	99.97%
Raw bases	15,443,015,700	16,382,223,000	15,248,869,500	87,637,462
Clean bases	14,191,332,303	14,971,313,169	14,051,356,877	12,943,399,200
Average depth on target	202.29×	213.84×	198.87×	114.67×
Target covered ≥20×	99.66%	99.74%	99.84%	98.45%
SNPs	52,144	52,017	51,802	131,250
InDels	8,008	8,042	8,012	25,598

SNPs, single nucleotide polymorphisms; InDels, insertions-deletions.
*The percentage of clean reads mapped to the human reference genome (GRCh37/hg19).

3 Supplementary References

Adzhubei, I., Jordan, D. M., and Sunyaev, S. R. (2013). Predicting functional effect of human missense mutations using PolyPhen-2. *Curr. Protoc. Hum. Genet*. Chapter 7, Unit7.20. doi:10.1002/0471142905.hg0720s76

Choi, Y., and Chan, A. P. (2015). PROVEAN web server: A tool to predict the functional effect of amino acid substitutions and indels. *Bioinformatics* 31, 2745–2747. doi:10.1093/bioinformatics/btv195

Hebsgaard, S. M., Korning, P. G., Tolstrup, N., Engelbrecht, J., Rouzé, P., and Brunak, S. (1996). Splice site prediction in Arabidopsis thaliana pre-mRNA by combining local and global sequence information. *Nucleic Acids Res*. 24, 3439–3452. doi:10.1093/nar/24.17.3439

Reese, M. G., Eeckman, F. H., Kulp, D., and Haussler, D. (1997). Improved splice site detection in Genie. *J. Comput. Biol*. 4, 311–323. doi:10.1089/cmb.1997.4.311

Steinhaus, R., Proft, S., Schuelke, M., Cooper, D. N., Schwarz, J. M., and Seelow, D. (2021). MutationTaster2021. *Nucleic Acids Res.* 49, W446–W451. doi:10.1093/nar/gkab266

Vaser, R., Adusumalli, S., Leng, S. N., Sikic, M., and Ng, P. C. (2016). SIFT missense predictions for genomes. *Nat. Protoc*. 11, 1–9. doi:10.1038/nprot.2015.123

Yuan, M., Guo, Y., Xia, H., Xu, H., Deng, H., and Yuan, L. (2021). Novel SCN5A and GPD1L variants identified in two unrelated Han-Chinese patients with clinically suspected Brugada syndrome. *Front. Cardiovasc. Med*. 8, 758903. doi:10.3389/fcvm.2021.758903

Yu, X., Yuan, L., Deng, S., Xia, H., Tu, X., Deng, X., et al. (2022). Identification of DNAH17 variants in Han-Chinese patients with left-right asymmetry disorders. *Front. Genet*. 13, 862292. doi:10.3389/fgene.2022.862292
